# Staying Home, Tweeting Hope: Mixed Methods Study of Twitter Sentiment Geographical Index During US Stay-At-Home Orders

**DOI:** 10.2196/45757

**Published:** 2023-07-24

**Authors:** Xinming Xia, Yi Zhang, Wenting Jiang, Connor Yuhao Wu

**Affiliations:** 1 School of Public Policy and Management Tsinghua University Beijing China; 2 Institute for Contemporary China Studies Tsinghua University Beijing China; 3 Chinese Society for Urban Studies Beijing China; 4 Interdisciplinary Programs Office The Hong Kong University of Science and Technology Hong Kong Hong Kong; 5 Urban Governance and Design Thrust The Hong Kong University of Science and Technology (Guangzhou) Guangzhou China; 6 Department of Management Science and Information Systems Oklahoma State University Stillwater, OK United States

**Keywords:** COVID-19, Twitter, stay-at-home orders, dynamics of public opinion, multiperiod difference-in-differences model

## Abstract

**Background:**

Stay-at-home orders were one of the controversial interventions to curb the spread of COVID-19 in the United States. The stay-at-home orders, implemented in 51 states and territories between March 7 and June 30, 2020, impacted the lives of individuals and communities and accelerated the heavy usage of web-based social networking sites. Twitter sentiment analysis can provide valuable insight into public health emergency response measures and allow for better formulation and timing of future public health measures to be released in response to future public health emergencies.

**Objective:**

This study evaluated how stay-at-home orders affect Twitter sentiment in the United States. Furthermore, this study aimed to understand the feedback on stay-at-home orders from groups with different circumstances and backgrounds. In addition, we particularly focused on vulnerable groups, including older people groups with underlying medical conditions, small and medium enterprises, and low-income groups.

**Methods:**

We constructed a multiperiod difference-in-differences regression model based on the Twitter sentiment geographical index quantified from 7.4 billion geo-tagged tweets data to analyze the dynamics of sentiment feedback on stay-at-home orders across the United States. In addition, we used moderated effects analysis to assess differential feedback from vulnerable groups.

**Results:**

We combed through the implementation of stay-at-home orders, Twitter sentiment geographical index, and the number of confirmed cases and deaths in 51 US states and territories. We identified trend changes in public sentiment before and after the stay-at-home orders. Regression results showed that stay-at-home orders generated a positive response, contributing to a recovery in Twitter sentiment. However, vulnerable groups faced greater shocks and hardships during the COVID-19 pandemic. In addition, economic and demographic characteristics had a significant moderating effect.

**Conclusions:**

This study showed a clear positive shift in public opinion about COVID-19, with this positive impact occurring primarily after stay-at-home orders. However, this positive sentiment is time-limited, with 14 days later allowing people to be more influenced by the status quo and trends, so feedback on the stay-at-home orders is no longer positively significant. In particular, negative sentiment is more likely to be generated in states with a large proportion of vulnerable groups, and the policy plays a limited role. The pandemic hit older people, those with underlying diseases, and small and medium enterprises directly but hurt states with cross-cutting economic situations and more complex demographics over time. Based on large-scale Twitter data, this sociological perspective allows us to monitor the evolution of public opinion more directly, assess the impact of social events on public opinion, and understand the heterogeneity in the face of pandemic shocks.

## Introduction

Stay-at-home orders, also referred to as shelter-in-place orders, safer-at-home orders, or lockdowns, have been a highly debated intervention strategy used to mitigate the spread of COVID-19 in the United States. Between March 7 to June 30, 2020, a total of 51 states and territories implemented stay-at-home or similar orders, affecting 2824 (around 87.3%) of 3233 US counties. The mandatory-for-all orders aimed to restrict residents' movements, work, education, gatherings, and general activities. Regarding public health, the stay-at-home orders met their original goal of saving the population from COVID-19 cases and deaths [1,2]. Meanwhile, the stay-at-home orders brought other benefits, such as reducing air pollution concentrations [3] by holding down population mobility [4,5]. These orders, however, have adverse effects. For instance, they increased screen time before bed and reduced total sleeping time, leading to worsened moods [6], reduced physical activity time, and greater weight gain [7,8]. The strong linkage between stay-at-home orders and mental health has been documented. Stay-at-home orders severely affected mental health, such as greater health anxiety, financial worry, sadness, loneliness, and a decrease in general mental health score [9-11]. The efficacy of stay-at-home orders varied spatially due to the differences in the timing of orders [12] and sociodemographic factors in different states [13]. How to customize the stay-at-home orders from the disaster management perspective is unknown. Overall, the stay-at-home orders profoundly affected the lives of individuals and communities.

Because of stay-at-home orders, many people were confined to their homes and unable to engage in their usual activities. They may have turned to social media as a way to stay connected and informed. Therefore, stay-at-home orders accelerated the massive use of web-based platforms, including social media [14]. Among social media, Twitter is preferably used by users to express their reactions to ongoing epidemics and related policies [15,16]. Twitter is a microblogging platform that allows users to send and receive short messages called “tweets.” Tweets are limited to 280 characters and can include text, images, and links. Twitter is known for its real-time, public nature and is often used by individuals and organizations to share news, opinions, and updates. Users can follow other users, hashtags, or topics to receive updates on their feeds and engage with others by liking, commenting on, or retweeting tweets. Twitter is available as a website and as a mobile app and is free to use. Twitter can be a valuable tool for studying public opinion because it allows researchers to collect large amounts of data in real time and at a low cost. Researchers can use Twitter to track how political debates evolve and are perceived [17,18], investigate how rumors and opinions spread [19,20], and test the validity of models of complex social behavior [21]. There are a number of efforts that have been used in addition to traditional medical testing to understand and track the spread of contagious diseases [22], such as influenza [23-25] and Ebola virus disease [26,27]. Specifically, numerous researchers have used Twitter to document the unprecedented COVID-19 pandemic [28-32]. Large-scale Twitter databases on COVID-19 have been constructed to facilitate further analyses on specific topics [29]. One of the key areas of investigation has been the fluctuation of sentiment during the COVID-19 pandemic [28,30,31]. Some studies further studied the polarization and politicization of public discourse about COVID-19 in the public sphere [33-35]. Furthermore, Twitter users have expressed their emotions regarding public reactions to the pandemic, such as vaccine hesitancy [36]. Analyzing the content of tweets and the interactions between users can provide valuable data and insights for public health decision-making, improve emergency response efforts, and enable better preparation for future COVID-19 variants and other public health emergencies [31].

A significant area of research involves the use of Twitter data to characterize public emotions during the COVID-19 pandemic effectively. Prior research has used a longitudinal sample of Twitter users to determine that lockdown measures were associated with increased aggression [37]. Another study used a similar approach to analyze the impact of Wuhan's lockdown, revealing that Weibo’s (Chinese Twitter) posts had higher valence and arousal levels [38]. Some research adopts similar indicators of public emotion as ours, the sentiment score. Insights from Singapore suggest that containment measures have differing effects on sentiment scores. Specifically, restrictions on public activities and travel were associated with decreased sentiment scores, while facial coverings had the opposite effect [39]. Furthermore, a global study found that countries with lockdown policies experienced a more positive change in sentiment [40]. Different from existing studies, our research used the sentiment score, the Twitter sentiment geographical index (TSGI) quantified from 7.4 billion geo-tagged tweets data, to analyze the dynamics of sentiment feedback on stay-at-home orders across the United States. Moreover, we examined differential feedback from vulnerable groups, including older people groups with underlying medical conditions, small and medium enterprises (SMEs), and low-income groups, using a multiperiod difference-in-differences (DID) regression model and moderated effects analysis.

The impact of COVID-19 and corresponding policy interventions varies between demographics and socioeconomic contexts. Our research highlights 3 areas of focus: age structure, medical care system, and vulnerable businesses. Older people, with a higher mortality rate, confront a more severe epidemic crisis [41,42]. Moreover, the threat to older people's mental health is also nonnegligible. Older people above 60 years are the most vulnerable age group regarding mental health when confronted with COVID-19 [42,43]. Even though the stay-at-home orders can slow the transmission, older people, as the most protected group, face more from social isolation, which causes more severe mental disorders [43,44]. The ecological effect of lockdown on older people's emotions, which mixes various social consequences, is still underexplored.

COVID-19 has imposed a significantly greater burden on national medical care, and hidden problems have begun to emerge. The availability and equality of medical care significantly impact the pandemic's consequences. For example, Black people have disproportionate COVID-19 cases and deaths due to long-standing inequities in the health care system. Medical care is thus called for reform as a lesson learned from the epidemic [45,46]. Mental health care also similarly exacerbates mental health disorders and emotional trauma [47]. The impact of stay-at-home orders is expected to differ depending on the regional health care system. Stay-at-home orders alleviate part of the system's pressure and, as a result, affect regional emotions. COVID-19 has multifaceted impacts, including economic impacts. Economic uncertainty caused by the pandemic severely impacts well-being, mental health, and emotional hazards [48]. SMEs are the most vulnerable economic actors that are struck by the pandemic. Under COVID-19, SMEs face a significant failure rate increase, leading to increased unemployment and financial risks [49,50]. We anticipate that COVID-19 policy intervention will independently influence economic resilience and vary depending on its economic structure. Regions with more vulnerable SMEs are expected to respond more strongly to policy intervention.

The COVID-19 pandemic has highlighted the critical role of public health interventions in mitigating the spread of airborne viruses. Stay-at-home orders have been shown to be effective in suppressing transmission [51,52], but they have also had side effects on mental health [9]. This study underscores the importance of a comprehensive understanding of the implications of such interventions. By exploring the interplay between public health measures and mental health outcomes, this research sheds light on the potential trade-offs between disease control and individual well-being. Such insights can inform future pandemic responses and help safeguard public health during crises. Consistent with previous research, we hypothesized that Twitter activity was a barometer of public perceptions of the pandemic and the stay-at-home orders. As the extent of these restrictions varies regionally, it is still being determined what impact the stay-at-home orders have had on tweet sentiment on a state-by-state basis. We, therefore, focused on quantifying daily Twitter sentiment scores in the United States during the implementation of the stay-at-home orders in each state by analyzing over 7.4 billion geo-tagged tweets. The main objective was to understand the dynamics of public opinion generated by the US stay-at-home orders and the differential feedback of sentiment on the stay-at-home orders from groups in different situations and backgrounds.

Our research makes 3 contributions to the literature. First, previous research has focused on the overall impact of lockdown policies but has overlooked potential regional variations in their effects. Our study identifies vulnerable regions and enhances current policy strategies. Second, in contrast to Twitter data characterized by keywords, our use of geo-tagged data enables us to accurately determine whether Twitter users were affected by lockdown orders in their respective locations, thereby improving precision in our analyses. Third, most studies to date have compared changes in sentiment scores in specific regions before and after lockdowns. In contrast, our research uses the multiperiod DID method, which effectively eliminates general trends due to time variation and isolates the causal effect of the stay-at-home orders on the treatment group (ie, states under orders).

## Methods

### Twitter Sentiment Geographical Index

The raw tweet data used to generate the global sentiment and geography index dataset comes from the Geotweet Archive (version 2.0), which is developed and maintained by the Harvard Center for Geographic Analysis [53]. The Archive is a global collection of geo-tagged tweets spanning time, geography, and language. It extends from 2010 to the present and is updated daily. Natural language processing techniques that include the Bidirectional Encoder Representations from the Transformers model developed by Google and the multilingual model (for handling 104 languages) are applied to a comprehensive archive of 7.4 billion geo-tagged tweets. Tweets are transformed into 768-dimensional text vectors and analyzed by a neural classifier to obtain single sentiment scores that indicate the positive or negative of the tweets. After that, the tweets are aggregated at different administrative levels (ie, country, state, and county levels) to calculate the TSGI. It primarily provides a detailed index of emotions across time (ie, a decade from 2012 to 2022) and geography (ie, 164 countries) with the sentiment classification accuracy of 83% [54]. To the best of our knowledge, it is the first subjective well-being data set at this scale and granularity.

### Model Design

To validate the relationship between stay-at-home orders and Twitter sentiment, this study constructs a multiperiod DID regression model (equation 1) at the state level, as different states adopt restrictive policies at different points in time. The following equation is estimated with robust standard errors.


*TSGI_it_ = β_0_ + β_1_Policy + β_2_Cases_it_ + γ_t_ + δ_i_ + ε_it_*
**(1)**

The dependent variable is the Twitter sentiment score in state *i* and day *t*. The score is a float value between 0 and 1, where 1 represents a positive sentiment, and 0 represents a negative sentiment. *Policy* represents the policy variable, which is 1 when the stay-at-home orders are in effect and 0 vice versa. *Cases_it_* includes control variables directly related to the pandemic [55,56], including the number of new cases and deaths. To avoid omitted variables, the model controls for time-fixed effects *γ_t_*, and state-fixed effects *δ_i_*. *ε_it_* is the error term. A robust is adopted. We pooled all observations from March 7 to June 30, 2020, as this is the period in which different states implemented stay-at-home orders that mandate residents to stay at home unless necessary.

The moderating effects of different attributes were further measured by constructing regression models with crossover terms (equation 2).


*TSGI_it_ = β_0_ + β_1_Policy × X_i_ + β_2_Cases_it_ + γ_t_ + δ_i_ + ε_it_*
**(2)**

To further explore heterogeneity, we built cross-variables based on the attributes of the different states, having explored the moderating effect of the different attributes. *X_i_* represents the different attribute characteristics of state *i*, including economic development, demographics, and vulnerable groups.

### Data Description

Data in this study are shown in Table 1. Our data cover 50 states and Washington, DC, in the United States from March 7 to June 30, 2020. Therefore, it includes 5916 observations (51 states and territories × 116 days) at the state-day level. Our key outcome variable is TSGI, with a mean score of 0.574, an SD of 0.016, and a range from 0 to 1. Higher TSGI indicates more positive sentiment in the state's geo-located Twitter. Our key independent variable is *Policy* (with 1 for in-effect stay-at-home orders and 0 for no stay-at-home orders). Our control variable includes the number of confirmed cases at the state level. In addition, the robustness test is conducted on the number of deaths. Other variables, most of which are used to explore the heterogeneity in the policy effect, can be divided into 3 categories: demographic features, including population size, sex ratio, and percent of the population aged 65 years and above; socioeconomic features, including income per capita, percentage of the homeless population, Gini index, and percent of SMEs; and health-related factors, including the cost of medical care, and hospital beds per 1000 people.

**Table 1:** Variables used in the model and analysis.

Variable	Description	Source	Observations, n	Mean (SD)	Range
TSGI^a^	Twitter sentiment geographical index	MIT^b^ Sustainable Urbanization Lab and Harvard's Center of Geographical Analysis	5916	0.574 (0.016)	0.633
Policy	Stay-at-home orders (1) or not (0)	Centers for Disease Control and Prevention, New York Times, manually collected by authors	5916	0.346 (0.475)	0-1
Cases	Number of new COVID-19 cases reported per day as a proportion of the total population in each state	New York Times	5842	0.003 (0.003)	0-0.019
Deaths	The total number of new deaths from COVID-19 reported, in logarithms	New York Times	5842	0.0001 (0.0002)	0-0.0016
lnElderly	Estimated percent of the population age is 65 years and older, 2016-2020, in logarithms	US Census	5916	2.79 (0.123)	2.405-3.026
Homeless	Homelessness in 2020 as a percentage of the total population of the states	The US Department of Housing and Urban Development	5916	0.001 (0.001)	0.0003-0.009
Medicalcare	Aggregate cost of medical care in 2019, per capita	PolicyMap and Quantitative Innovations	5916	7208.149 (508.305)	5737.715-8462.92
SMEs^c^	Percent of firms less than 3 years old in 2019	Census Business Dynamics Statistics	5916	18.020 (2.843)	13.23-24.64
Gini	Estimated inequality of household income according to the Gini index, 2016-2020	US Census	5916	0.465 (0.020)	0.42-0.52
lnHospitalbeds	The rate of hospital beds per 1000 people in 2016, in logarithms	Health Resources and Services Administration	5916	1.105 (0.261)	0.641-1.835
lnIncome	Estimated per capita income, 2016-2020, in logarithms	US Census	5916	10.449 (0.154)	10.144-10.979
lnPopulation	Population in 2020, in logarithms	US Census	5916	4.652 (1.520)	0.246-9.330
Sexratio	Estimated ratio of male population to female population, 2016-2020	US Census	5916	97.686 (3.281)	90-109

^a^TSGI: Twitter sentiment geographical index.

^b^MIT: Massachusetts Institute of Technology.

^c^SME: small and medium enterprises.

### Ethical Considerations

This study did not require research ethics approval, as the utilized data was accessible to the public. The TSGI dataset, acting as a subjective well-being index, does not incorporate any specific Twitter user's handle or the content of their tweets. In terms of geography, the TSGI data is compiled at the county level, ensuring that the geo-tweets contributing to it remain untraceable to individual users. Other datasets in Table 1 are aggregated. Additionally, there was no engagement between the study's authors and any Twitter users.

## Results

### COVID-19 and Stay-at-Home Orders

**Figure 1:**
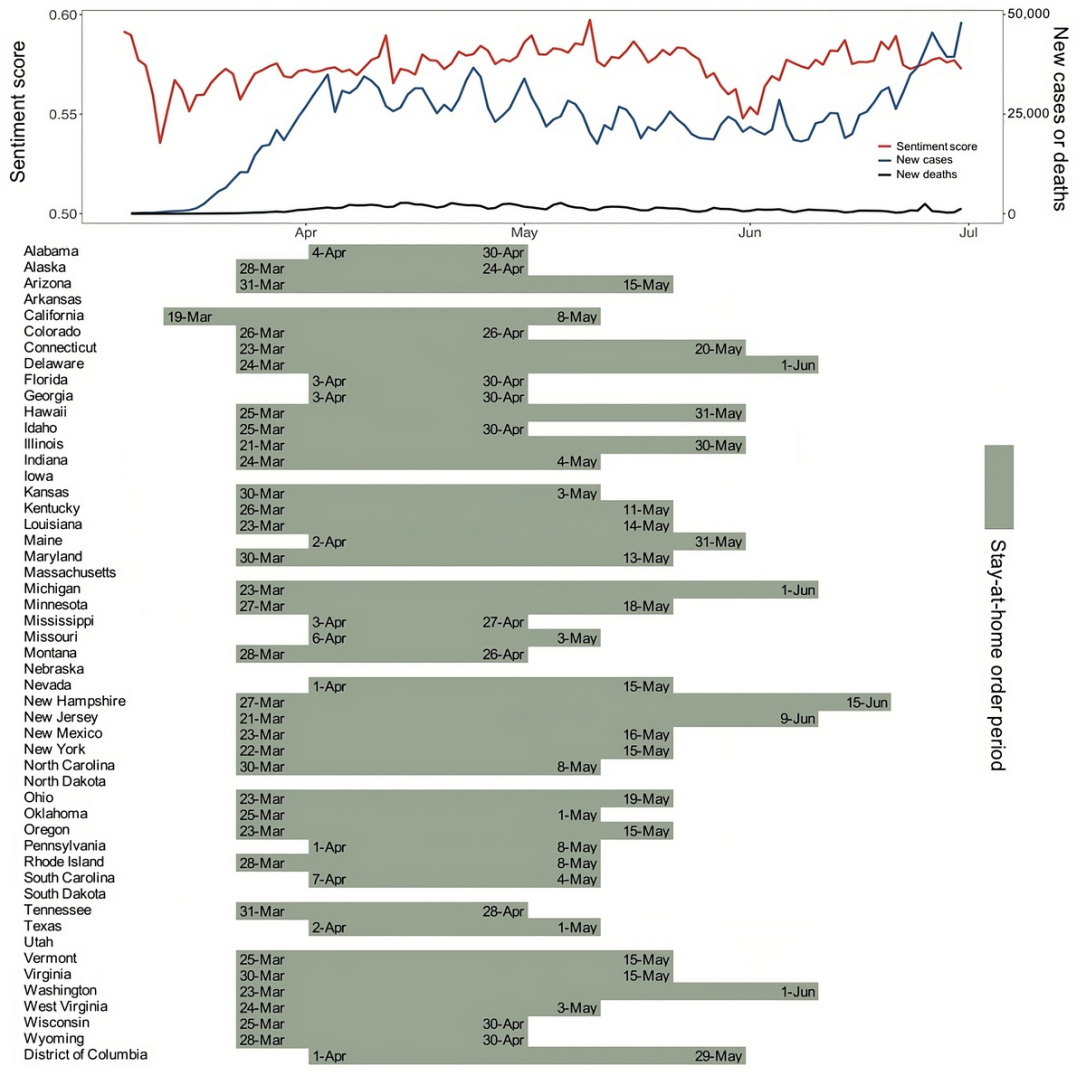
Confirmed cases, deaths, local stay-at-home orders, and Twitter sentiment geographical index in the United States.

Figure 1 depicts a detailed overview of daily cases, deaths, sentiment, and stay-at-home orders. Until the first week of April 2020, the number of daily new cases increased dramatically. In response to the worsening pandemic situation, most states had adopted stay-at-home orders by the end of March. The policy implementation also increased the sentiment index. While the number of new cases remained high in late April, some states have relaxed their restrictions. The orders were lifted in late May in most states.

### Sensitivity Fluctuations Before and After the Policy

**Figure 2:**
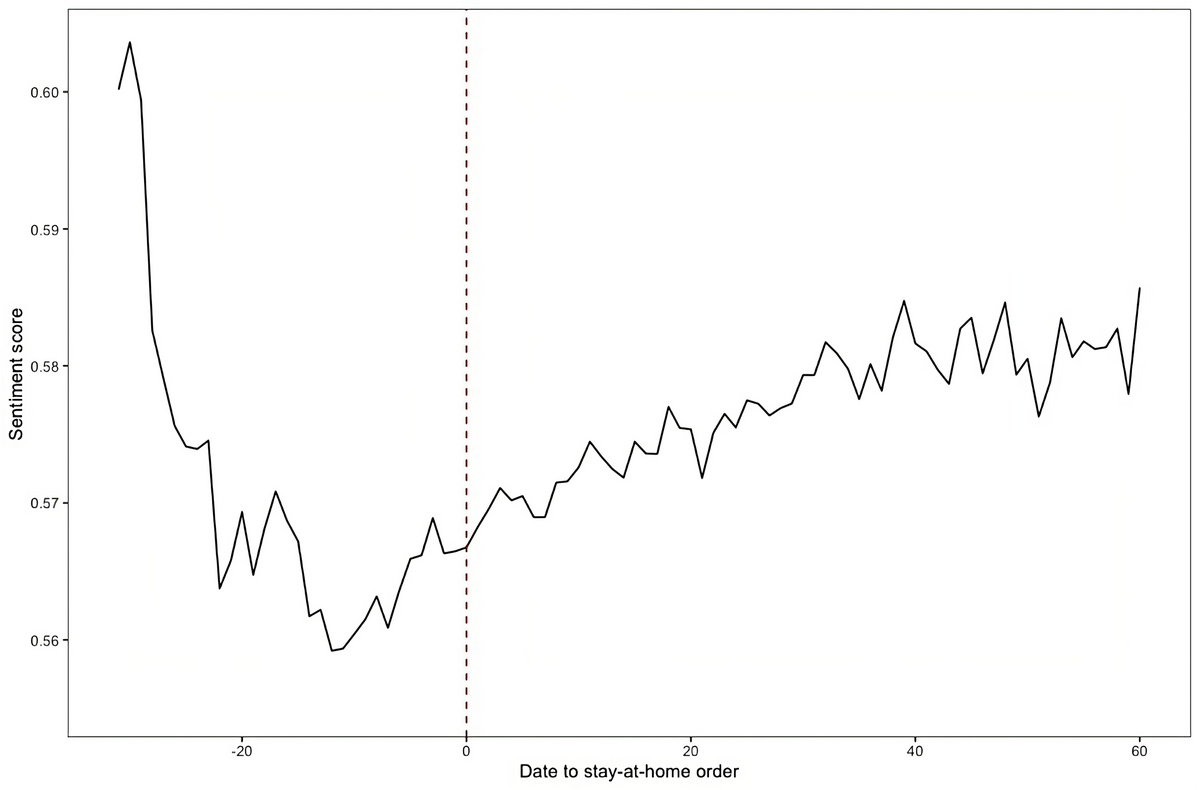
Sensitivity fluctuations before and after the policy. Only states with stay-at-home orders are included.

The anticipation and implementation of policy interventions have a positive influence on emotions and help in the recovery of public sentiments. Figure 2 shows that sentiment scores dropped dramatically initially (from 0.603 to 0.575). It may come from the fear and worry about the newly emerged COVID-19, which spread quickly and widely. Before the lockdown, we can observe some recovery in the sentiment. At the commencement of stay-at-home orders, the sentiment score increased from 0.559 to 0.566. Furthermore, the score continued to rise after stay-at-home orders, reaching 0.572, 0.579, and 0.585 at 10, 30, and 60 days, respectively.

### Overview: Mobility Restriction Measures Generate a Positive Response on Twitter

The model in Table 2 shows the change in the degree of impact of the policy on the TSGI over time. The policy has a significant positive effect on Twitter sensitivity, both a 3-day lag (approximately 5.7% increase) and a 7-day lag (approximately 9.1% increase), indicating that the mobility restriction measures generated a positive response on Twitter. This is broadly consistent with the trend shown in Figure 2. At a lag of 14 days, the policy effect is insignificant, which shows the policy has been limited over time. Meanwhile, the number of COVID-19 confirmed cases was negatively associated with Twitter sentiment.

**Table 2:** Regression results of policy effects.

	Dependent variable: TSGI^a^							
	(1)			(2)			(3)	
	Timelag_3	*P* value	Timelag_7		*P* value	Timelag_14		*P* value
Policy	0.057	.09	0.091		.008	−0.042		.27
Cases	–12.695	.01	−12.986		.01	−8.097		.16
State FE^b^	Yes		Yes			Yes		
Day FE	Yes		Yes			Yes		
Constant	56.141	<.001	57.777		<.001	57.803		<.001
Observations	5048		4933			4242		
*R* ^2^	0.827		0.819			0.802		

^a^TSGI: Twitter sentiment geographical index.

^b^FE: fixed effect.

### Vulnerable Groups Face Shocks and Hardships

The regression results for the moderating effects of different attributes in various states are presented in Tables 3 and 4, respectively. The cross-sectional variables of older people 65 years or older, medical care costs, SMEs, and policy were significantly negative. States with many vulnerable groups, such as older people, those with high underlying diseases, and SMEs, faced shocks and hardships during the COVID-19 pandemic. This finding is consistent with previous findings. For instance, previous research found that self-isolation caused by stay-at-home orders would disproportionately affect older individuals whose only social contact is out of the home, such as at daycare venues, community centers, and places of worship [44]. Other findings show that the high-risk population included 70 years or older or those with at least 1 underlying condition [57]. The most hazardous comorbidities in fatal cases were hypertension, diabetes, and cardiovascular diseases [58].

**Table 3:** Regression results for disadvantaged groups.

	Dependent variable: TSGI^a^							
	(4)			(5)			(6)	
	Timelag_3	*P* value	Timelag_3		*P* value	Timelag_3		*P* value
Policy	2.390	<.001	1.069		.002	0.407		.004
Cases	−9.745	.06	−11.953		.02	–11.755		.02
Policy*lnElderly	−0.830	<.001	—^b^		—	—		—
Policy*Medicalcare	—	—	−0.000		.004	—		—
Policy*SMEs^c^	—	—	—		—	−0.020		.009
Control FE^d^	Yes		Yes			Yes		
State FE	Yes		Yes			Yes		
Day FE	Yes		Yes			Yes		
Constant	56.133	<.001	56.132		<.001	56.156		<.001
Observations	5048		5048			5048		
*R* ^2^	0.828		0.827			0.827		

^a^TSGI: Twitter sentiment geographical index.

^b^Not available.

^c^SME: small and medium enterprises.

^d^FE: fixed effect.

**Table 4:** Regression results for moderating effects.

	Dependent variable: TSGI^a^																					
	(7)		(8)			(9)			(10)			(11)			(12)			(13)			(14)	
	Timelag_3	*P* value	Timelag_7	*P* value	Timelag_3		*P* value	Timelag_7		*P* value	Timelag_3		*P* value	Timelag_7		*P* value	Timelag_3		*P* value	Timelag_7		*P* value
Policy	1.48	.26	3.591	.004	0.111		.02	0.403		.001	0.737		.33	1.574		<.001	−0.783		.45	−2.387		.006
Cases	−13.713	.01	−15.357	.004	−13.505		.01	−16.682		.006	−13.088		.009	−14.507		.002	−13.624		.009	−15.191		.004
Policy*lnIncome	−0.136	.27	−0.335	.004																		
Policy*Gini					−1.455		.26	−3.188		.001												
Policy*lnPopulation											−0.011		.58	−0.064		<.001						
Policy*Sexratio																	0.009		.42	0.025		.005
Control FE^b^	Yes		Yes		Yes			Yes			Yes			Yes			Yes			Yes		
State FE	Yes		Yes		Yes			Yes			Yes			Yes			Yes			Yes		
Day FE	Yes		Yes		Yes			Yes			Yes			Yes			Yes			Yes		
Constant	56.145	<.001	57.803	<.001	56.144		<.001	57.791		<.001	56.144		<.001	57.784		<.001	56.143		<.001	57.770		<.001
Observations	5048		4933		5048			4933			5048			4933			5048			4933		
*R* ^2^	0.827		0.819		0.827			0.82			0.827			0.819			0.827			0.819		

^a^TSGI: Twitter sentiment geographical index.

^b^FE: fixed effect.

### The Moderating Effect of Economic and Demographic Characteristics

Table 4 reports the regression results for introducing cross terms with a lag of 3 and 7 days, respectively. Both present the emergence of a moderating effect of different attribute characteristics on the policy-sentiment relationship over time. Regarding economic characteristics, the issue of per capita income comes to the fore, with the cross-variable significantly negative at a lag of 7 days, creating an effect on the positive policy-sentiment impact. Similarly, the Gini coefficient, which reflects the level of imbalance, also exhibits a dampening effect. From a demographic perspective, provinces with high population density have a negative impact. A high male-to-female sex ratio means more males and relatively negative policy feedback, which may be due to the fact that they might have higher expression of angiotensin-converting enzyme 2 regulated by male sex hormones for higher COVID-19 infection and poor clinical outcomes [59-61].

### Robustness Tests

The robustness of the model was tested through different time lag effects and substituting variables. First, given that the emotional impact of fatal cases is more direct than that of confirmed cases, with a high mortality rate of 14% in April 2020 [62], the US population is generally more concerned about deaths. Therefore, this paper uses the number of death cases to replace confirmed cases, taking the logarithm of the number of death cases to bring into the model. The results remain almost identical to those of the base model. Second, we examined similarly different time-lagged effects, and these time-varying patterns are retained and do not alter the main findings. The regression results of the robustness tests are reported in Tables 5 and 6.

A state in the United States can be very heterogeneous, with a significant disparity in policy implementation and social demographics. Although stay-at-home orders policy variation exists primarily at the state level, it is necessary to conduct county-level analysis to further validate our results. However, many indicators of concern in this study are not available at the county level. Still, we collated the available county-level data on stay-at-home orders, the number of confirmed cases and deaths, older people, population, and hospital beds and rematched them with the county-scale Twitter sentiment indicators as a robustness test (see Table 7). The results at the county level remain consistent with those from the baseline regressions at the state level in Tables 2, 3, and 6 so that the main findings are further validated.

**Table 5:** Robustness tests of the impact of policy on TSGI^a^ (disadvantaged groups).

	Dependent variable: TSGI																																		
	(15)				(16)					(17)					(18)					(19)					(20)					(21)				(22)	
	Timelag_3	*P* value		Timelag_7			*P* value		Timelag_3			*P* value		Timelag_3			*P* value		Timelag_3			*P* value		Timelag_7			*P* value		Timelag_7			*P* value	Timelag_7		*P* value
Policy	0.018	.60		0.080			.02		2.655			<.001		1.309			<.001		0.283			.06		1.311			.01		0.779			.01	0.199		.27
lnDeaths	−0.091	<.001		−0.075			.002		−0.083			.001		−0.090			<.001		−0.087			.001		−0.076			.002		−0.079			.001	−0.072		.002
Policy*lnElderly									−0.938			<.001												−0.443			.02								
Policy*Medicalcare														−0.001			<.001												−0.001			.02			
Policy*SMEs^b^																			−0.015			.05											−0.007		.43
Control FE^c^	Yes			Yes					Yes					Yes					Yes					Yes					Yes				Yes		
State FE	Yes			Yes					Yes					Yes					Yes					Yes					Yes				Yes		
Day FE	Yes			Yes					Yes					Yes					Yes					Yes					Yes				Yes		
Constant	56.693	<.001		58.277			<.001		56.651			<.001		56.684			<.001		56.686			<.001		58.270			<.001		58.294			<.001	58.266		<.001
Observations	4537			4447					4537					4537					4537					4447					4447				4447		
*R* ^2^	0.826			0.817					0.827					0.827					0.826					0.817					0.817				0.817		

^a^TSGI: Twitter sentiment geographical index.

^b^SME: small and medium enterprises.

^c^FE: fixed effect.

**Table 6:** Robustness tests of TSGI^a^ on key features.

	Dependent variable: TSGI														
	(23)			(24)			(25)		(26)		(27)			(28)	
	Timelag_7	*P* value	Timelag_7		*P* value	Timelag_7		*P* value	Timelag_7	*P* value	Timelag_7	*P* value	Timelag_7		*P* value
Policy	−0.152	.17	2.579		.05	0.342		.001	−1.719	.06	0.049	.23	1.292		.009
lnDeaths	−0.071	.003	−0.075		.002	−0.072		.003	−0.073	.002	−0.077	.001	−0.070		.004
Policy*lnHospitalbeds	0.206	.04													
Policy*lnIncome			−0.239		.05										
Policy*lnPopulation						−0.054		.002							
Policy*Sexratio									0.019	.05					
Policy*Homeless											20.274	.09			
Policy*Gini													−2.602		.01
Control FE^b^	Yes		Yes			Yes			Yes		Yes		Yes		
State FE	Yes		Yes			Yes			Yes		Yes		Yes		
Day FE	Yes		Yes			Yes			Yes		Yes		Yes		
Constant	58.260	<.001	58.289		<.001	58.244		<.001	58.249	<.001	58.290	<.001	58.235		<.001
Observations	4447		4447			4447			4447		4447		4447		
*R* ^2^	0.817		0.817			0.818			0.817		0.817		0.817		

^a^TSGI: Twitter sentiment geographical index.

^b^FE: fixed effect.

**Table 7:** County-level robustness tests.

	Dependent variable: TSGI_county^a^									
	(29)		(30)			(31)			(32)	
	Timelag_3	*P* value	Timelag_3	*P* value	Timelag_7		*P* value	Timelag_7		*P* value
Policy	3.422	<.001	21.31	<.001	2.354		.45	−3.194		.34
Cases	−0.013	.01	−0.0154	.02	−1.889		<.001			
Policy*lnElderly			−6.256	<.001						
Policy*lnPopulation					−0.390		.07			
lnDeaths								−0.953		.004
Policy*lnHospitalbeds								3.626		.10
Control FE^b^	Yes		Yes		Yes			Yes		
Constant	56.24	<.001	56.258	<.001	59.496		<.001	59.145		<.001
Observations	26,923		26,923		26,923			26,923		
*R* ^2^	0.308		0.143		0.380			0.356		

^a^TSGI: Twitter sentiment geographical index.

^b^FE: fixed effect.

## Discussion

### Principal Results

This study investigated the effects of stay-at-home orders on geo-tweet sentiments in the United States. Results showed several essential points. First, the anticipation and implementation of stay-at-home orders positively influenced discussions on Twitter and helped in the recovery of tweet sentiments. Second, the stay-at-home orders' positive effect on tweet sentiments was significant at both a 3-day lag and a 7-day lag and became insignificant at 14 days. Third, vulnerable groups, such as older people, those with high underlying diseases, and SMEs, faced shocks and hardships during the COVID-19 pandemic. Fourth, in states with low incomes, large gaps between rich and poor, high population densities, and large gender ratios, the positive effects of stay-at-home orders on public sentiment are weakened.

### Comparison With Previous Research

This study provides a sociological perspective on the emotional feedback from ordinary people to understand the impact of COVID-19 and stay-at-home orders on different groups. Previous studies on social media Twitter examining public opinion on COVID-19 in different countries, mostly through keyword clustering, lacked further quantification of text mining, a gap that TSGI bridges. More meaningfully, in line with biological and pharmaceutical findings of more significant impact on older age groups and underlying diseases groups, this study finds through a sociological lens that COVID-19 has a large impact on states with a large proportion of vulnerable groups, including older people, underlying diseases, and even on startup businesses.

Our results show fluctuations before and after the implementation of stay-at-home orders. It is consistent with previous findings, which show that the spread of the infection caused fear-related sentiments in the early stage of the pandemic, and then people expressed support for stay-at-home orders [14]. With the continued evolution of the pandemic, the portion of positive Tweets remained level, and that of negative Tweets rose, indicating that pandemic fatigue, stress, and loneliness started taking a toll on how people felt about stay-at-home orders.

In the results on the effects of stay-at-home orders in the different lag of days, we found that the policy effects were insignificant at a lag of 14 days, indicating the policy efficacy faded over time. There is a debate on defining when to “exit strategy,” lift orders, or reopen. Some researchers suggested that a “localization” strategy is efficient after 14 days of lockdown [63], while others found that the efficacy of lockdown measures worldwide continued to grow up to 20 days after implementation [12]. Our results confirmed the latter one and specified that the efficiency of stay-at-home orders was up to 14 days in the United States.

### Limitations

There are limitations to our study. First, Twitter users are not representative of the population as a whole, and the sentiment of tweets only indicates the opinions and concerns of web-based users, making it impossible to avoid sampling bias in web-based data. However, the Twitter data set remains a valuable resource with a broad audience as a US-based social media, allowing us to study the real-time reactions of Twitter users and web-based activity related to COVID-19 [31]. Surprisingly, this relatively young, web-based-available, and expressive group still responds significantly to the feedback on COVID-19 in states with a high proportion of older people. Second, our study draws primarily on the TSGI, a quantitative sentiment value, but this is for users of Twitter in the United States. Still, its quantification may need to be more readily generalizable to non–English-speaking countries, such as China and Japan, where language differences can make the quantification of text mining difficult. In this way, future research should compare with findings in Weibo (ie, Twitter in China) and Twitter in other languages. Third, this paper uses states as the unit of study based on the general administrative scope of policy development and implementation. However, a state in the United States can be very heterogeneous, with significant variation in policy implementation and sociodemographics. Although stay-at-home orders are implemented at the state level, the ultimate performance is at the city or county level, so we further conducted robustness tests using county-level data (Table 7). In fact, COVID-19 has a differentiated impact on cities with high population density and rural areas with inadequate medical care, and further distinctions need to be made in future research.

### Conclusions

We examined the evolution of public opinion regarding stay-at-home orders in the United States during the COVID-19 pandemic through TSGI, measured based on 7.4 billion geo-tagged tweets. We found a clear positive shift in public opinion about COVID-19, with this positive impact occurring mainly after stay-at-home orders. However, this positive sentiment is time-limited, with 14 days later having allowed people to be more influenced by the status quo and trends. At the same time, feedback on the stay-at-home orders is no longer positively significant. In particular, negative sentiment is more likely to be generated in states with a large proportion of vulnerable groups, and the policy plays a limited role. The pandemic hits older people, those with underlying diseases, and SMEs very directly and hurts the states with cross-cutting economic situations and more complex demographics as the stay-at-home orders implemented over time. Based on large-scale Twitter data, this sociological perspective allows us to monitor the evolution of public opinion more directly, assess the impact of social events on public opinion, and understand the heterogeneity in the face of pandemic shocks. The results of this study can provide guidance for policy makers and public health officials to effectively respond to future pandemics while considering the diverse needs and situations of different groups.

## Abbreviations

DID: difference-in-differences
SME: small and medium enterprises
TSGI: Twitter sentiment geographical index

## Data Availability

Our data sets mainly have three parts: (1) data set about the Twitter sentiment geographical index can be accessed from the Center of Geographical Analysis at Harvard University [54] (2) data sets about variables used in the model and analysis (which are listed in Table 1) can be accessed from PolicyMap [64]; and (3) the data sets about the numbers of confirmed cases and deaths due to COVID-19 can be accessed from *New York Times* [65].
